# Cadherins Interact With Synaptic Organizers to Promote Synaptic Differentiation

**DOI:** 10.3389/fnmol.2018.00142

**Published:** 2018-04-30

**Authors:** Masahito Yamagata, Xin Duan, Joshua R. Sanes

**Affiliations:** Center for Brain Science and Department of Molecular and Cellular Biology, Harvard University, Cambridge, MA, United States

**Keywords:** adhesion, cadherin, HEK293, LRRtm2, N-cadherin, neurexin, neuroligin, synaptogenesis

## Abstract

Classical cadherins, a set of ~20 related recognition and signaling molecules, have been implicated in many aspects of neural development, including the formation and remodeling of synapses. Mechanisms underlying some of these steps have been studied by expressing N-cadherin (*cdh2*), a Type 1 cadherin, in heterologous cells, but analysis is complicated because widely used lines express *cdh2* endogenously. We used CRISPR-mediated gene editing to generate a Human embryonic kidney (HEK)293 variant lacking Cdh2, then compared the behavior of rodent cortical and hippocampal neurons co-cultured with parental, *cdh2* mutant and *cdh2*-rescued 293 lines. The comparison demonstrated that Cdh2 promotes neurite branching and that it is required for three synaptic organizers, neurologin1 (NLGL1), leucine-rich repeat transmembrane protein 2 (LRRtm2), and Cell Adhesion Molecule 1 (Cadm1/SynCAM) to stimulate presynaptic differentiation, assayed by clustering of synaptic vesicles at sites of neurite-293 cell contact. Similarly, Cdh2 is required for a presynaptic organizing molecule, Neurexin1β, to promote postsynaptic differentiation in dendrites. We also show that another Type I cadherin, Cdh4, and a Type II cadherin, Cdh6, can substitute for Cdh2 in these assays. Finally, we provide evidence that the effects of cadherins require homophilic interactions between neurites and the heterologous cells. Together, these results indicate that classical cadherins act together with synaptic organizers to promote synaptic differentiation, perhaps in part by strengthening the intracellular adhesion required for the organizers to act efficiently. We propose that cadherins promote high affinity contacts between appropriate partners, which then enable synaptic differentiation.

## Introduction

N-cadherin (Cdh2) has been implicated in many early steps in neural development including formation of the neural tube, neuronal migration, and axon elongation (Hirano and Takeichi, 2012). The observation that Cdh2 is concentrated at synaptic sites (Yamagata et al., 1995; Fannon and Colman, 1996; Uchida et al., 1996; Benson and Tanaka, 1998) raised the possibility that it also plays roles in the formation and function of synapses. These roles have been difficult to study *in vivo*, however, for two reasons. First, *cdh2* null mutant mice die embryonically, prior to formation of functional neural circuits. Second, many if not most central neurons express other classical cadherins (e.g., Fannon and Colman, 1996; Miskevich et al., 1998; Yamagata et al., 2006; Williams et al., 2011; Duan et al., 2014; Kuwako et al., 2014; reviewed in Redies, 2000; Hirano and Takeichi, 2012), which may compensate for loss of Cdh2.

As an alternative, several groups have turned to analysis of Cdh2-deficient neurons in culture. Several sources have been used, including neurons derived from *cdh2*-null embryonic stem cells (Jüngling et al., 2006; Stan et al., 2010) or from a conditional *cdh2* mutant (Kadowaki et al., 2007), neurons in which *cdh2* expression was attenuated with shRNA (Aiga et al., 2011), and neurons in which Cdh2 function was inhibited by introduction of a dominant negative construct (Togashi et al., 2002; Bozdagi et al., 2004) or application of function blocking antibodies (Inoue and Sanes, 1997; Bozdagi et al., 2000). In each case, loss of cadherin led to synaptic defects, but results differed from report to report, perhaps because of the different systems and assays employed.

Here, as an alternative approach, we expressed cadherins and synaptic organizing proteins in heterologous cells, cultured them with cortical or hippocampal neurons, and assessed synaptic differentiation of neurites that encountered the transfected cells. Our particular interest was in testing the possibility that cadherins interact with synaptic organizing molecules, proteins that act transsynaptically to promote pre- and postsynaptic differentiation (Yogev and Shen, 2014; de Wit and Ghosh, 2016). Of these, the best studied are neurexins (NRXNs) and neuroligins (NLGNs), which are concentrated in pre- and postsynaptic membranes, respectively, at many and possibly most mammalian central synapses (Bemben et al., 2015; Südhof, 2017). Their ability to promote synaptic differentiation was first revealed by culturing neurons with nonneural cells engineered to express one or the other. Synaptic vesicles formed clusters in regions of neurites that contacted NLGN-expressing cells, and they were capable of releasing neurotransmitter when stimulated (Scheiffele et al., 2000; Fu et al., 2003; Biederer and Scheiffele, 2007). Conversely, neurotransmitter receptors in dendrites aggregated at sites that contacted nonneural cells engineered to express NRXNs (Graf et al., 2004).

Two previous reports have used neurons in which *cdh2* was down-regulated or deleted to provide evidence that Cdh2 and NLGN interact to promote presynaptic differentiation (Stan et al., 2010; Aiga et al., 2011). To extend this work, we aimed to ask: (1) Can Cdh2 promote presynaptic differentiation on its own? (2) Is it required for NLGN-dependent presynaptic differentiation in the absence of other synaptic components? (3) If so, does its role require its adhesive function and/or its ability to signal? (4) Is the requirement specific for NLGN or is it shared by other synaptic organizers? (5) Can other classical cadherins promote presynaptic differentiation on their own or in collaboration with NLGN? (6) Do postsynaptic roles of cadherins require a corresponding presynaptic cadherin to which it can bind homophilically? and (7) Can cadherins promote postsynaptic differentiation, either along or together with NRXN?

We planned to answer these questions using the Human embryonic kidney (HEK) cell line, HEK293, which is easy to manipulate and has been used in other studies on synaptogenesis (e.g., Scheiffele et al., 2000; Fu et al., 2003; Biederer and Scheiffele, 2007). However, HEK293 cells and COS cells, which are also frequently used for such studies express *cdh2* endogenously (Flannery and Brusés, 2012), complicating interpretation of experiments that involve introduction of exogenous cadherins. We therefore used CRISPR/Cas9-dependent mutagenesis to derive a *Cdh2*-null subclone, which we used for our studies. This line may be generally useful for studies of adhesive interactions (e.g., Goodman et al., 2016).

## Materials and Methods

### Animals

Mice and rats were used in accordance with NIH guidelines and protocols approved by Institutional Animal Use and Care Committee at Harvard University. No human subjects were used. Pregnant CD1 mice were purchased from Jackson, or produced in house. Pregnant Sprague-Dawley rats were purchased from Charles River.

To generate *Cdh6;cdh9;cdh10* triple mutant mice, we first generated *cdh6* and *cdh10* mutants by targeted insertion of a frt-neo-frt cassette, a 6xmyc-tagged CreER-T2, and polyadenylation signal at the translational start site of the *cdh6* and *cdh10* coding sequence, accompanied by deletion of their predicted signal sequences. The *cdh6* line was described previously (Kay et al., 2011), and is available from Jackson Laboratories (JAX mouse # 029428). Immunohistochemistry demonstrated that both were null alleles. The *cdh6* and *cdh10* genes are separated by <6 Mb in the same cadherin gene cluster on chromosome 15, but we were able to generate *cdh6;cdh10* double mutants by breeding. Because the *cdh9* gene is between the *cdh6* and *cdh10* genes, we deleted it by CRISPR-based genome editing in the double mutant background. We identified pups carrying large indels in the first coding exon of *cdh9*, which led to production of a short, truncated protein. Detailed methods and analysis of the triple mutant will be described elsewhere (Duan et al., submitted).

### 293 Cells

HEK-293T were purchased from the American Type Culture Collection (ATCC; Manassas, VA, USA). Since HEK-293T cells are occasionally reported to be contaminated with HeLa cells (International Cell Line Authentication Committee)^1^, we confirmed that the cells were G418 resistant, a characteristic of HEK-293T but not HeLa cells. Cells were cultured in Dulbecco Modified Eagle Medium supplemented with 10% (v/v) fetal calf serum and penicillin/streptomycin plus Normocin and G418 (Invivogen, San Diego, CA, USA; DMEM10). From this original culture, we isolated a subclone, 293TA, that grew well and was easy to subclone and transfect 293T and 293TA cells were indistinguishable in morphology and aggregation properties.

To mutate the *cdh2* gene in 293TA cells, a double strand DNAs corresponding to GCCGGATAGCGGGAGCGCTG flanked by a protospacer adjacent motif (PAM) sequence (Supplementary Figure S1A) was cloned into pX330 bearing *Staphylococcus aureus* Cas9 cDNA (Cong et al., 2013) to overexpress the corresponding short guide RNA under a U6 promoter. pX330 was obtained from Addgene (Cambridge, MA, USA). The plasmid was transfected into 293TA with DMRIE-C and OptiMEM (ThermoFisher/Invitrogen), and plated into 10 cm tissue culture dishes. Colonies were picked up, and expanded. To sequence the targeted *cdh2* sequences, the genes were amplified by PCR using primers CGTTTCTCCG CGCCGCTGTT and ACCGCCGCGTACCTGAAGCA. The amplified fragments were cloned into pCR8-TOPO vectors (ThermoFisher/Invitrogen), and selected with spectinomycin. Plasmid DNA was prepared from multiple colonies and sequenced. The 293NC line bearing null mutations of both alleles of *cdh2* was expanded and used in subsequent experiments.

To generate 293TA and 293NC lines stably expressing adhesion and recognition molecules, the sequences were amplified with Q5 DNA polymerase (New England Biolabs, Ipswich, MA, USA), digested with restriction enzymes, and cloned into a piggyBac transposon vector pXL-CAG-Zeocin-3xF2A. The cDNA was cloned after three tandemly-connected copies of a codon-optimized foot-and-mouth disease 2A segment, which was preceded by a signal sequence. The plasmid was cotransfected with a helper plasmid encoding piggyBac transposase (pCAG-mPBorf, Yamagata and Sanes, 2012). After selection with 1 mg/mL Zeocin (Invivogen, San Diego, CA, USA), >50% of stable transfectants expressed the inserted cDNA. This method appears superior to the conventional method, which usually yields <5% of high-expressing stable clones (also see Goodman et al., 2016; Martell et al., 2016). Surviving colonies were transferred to new plates and screened with antibodies to the protein product of the encoded cDNA. These cells were maintained in DMEM10.

The following cDNA sequences were generated by PCR, using high-fidelity Q5 DNA polymerase (NEB) and appropriate primers: (1) Cdh2, chicken N-cadherin originally obtained from M. Takeichi (Hatta et al., 1988; Miyatani et al., 1989); (2) Cdh2EC, a chicken cdh2 mutant that retained the extracellular and transmembrane domains, as well as the cytoplasmic juxtamembrane snorkeling motif involved in membrane insertion (Strandberg and Killian, 2003; Kim et al., 2011) but lacked the remainder of the cytoplasmic domain; (3) chicken Cdh2Cyt appended with a HA tag at N-terminal; (4) mouse neural cell adhesion molecule (NCAM) corresponding to NCAM180; (5) mouse NLGN1; (6) mouse NLGN1ΔPDZ; (7) mouse NRXN1β; (8) mouse LRRtm2; and (9) mouse Cdm1. Constructs 4–9 were cloned from adult mouse brain cDNA.

### Co-cultures of 293 Cells and Neurons

Neurons from E17–18 mouse cortex and P0–1 rat hippocampus were prepared using a Papain dissociation system (Worthington Biochemical Co., Lakewood, NJ, USA) according to the manufacturer’s protocol, and cultured on Matrigel (ThermoFisher/Invitrogen, Waltham, MA, USA, USA)-coated glass coverslips (Bellco Glass, Vineland, NJ, USA) in Neurobasal with B27 suppplement (ThermoFisher/Invitrogen) and Normocin (Invivogen, San Diego, CA, USA). For studies with mutants, cortical cultures were prepared separately from each embryo, and genotyped. Heterologous cells were transfected and cultured in advance and added to the culture as described by Biederer and Scheiffele (2007). After coculturing for 2 days, the coverslips were fixed with 4% (w/v) paraformaldehyde/PBS for analysis.

### RT-PCR

Total RNAs were purified using an illustra RNAspin Mini Kit (GE Healthcare, Boston, MA, USA). cDNA was synthesized with SuperScript III First-Strand Synthesis SuperMix for qRT-PCR (Invitrogen) from 1 μg of total RNA with or without the reverse transcriptase mixture. After RNaseH digestion, the synthesized cDNA was subjected to PCR (94°C, 2 min; 40 cycles of 94°C, 30 s/65°C, 30 s/72°C, 30 s and 72°C, 5 min) using EconoTaq DNA polymerase (Lucigen, Middleton, WI, USA). The primer sets used for amplification were as follows:

Primers for the deleted 5′ segment of human Cdh2 mRNA
Forward: GGATAGCGGGAGCGCTGCGGACC
Reverse: TTCACATTGAGAAGAGGCTGTCC
Primers for human GAPDH mRNA
Forward: AGGGCTGCTTTTAACTCTGGT
Reverse: CCCCACTTGATTTTGGAGGGA

### Cell Aggregation Assay

Stably-transfected or wild type 293TA or 293NC cells were labeled with Green Cell Tracker (ThermoFisher/Invitrogen), and trypsinized in 0.05% (w/v) trypsin (ThermoFisher/Invitrogen)/Hanks’ balanced salt solution supplemented with 20 mM HEPES, pH 7.4 (HBSS) in the presence of 1 mM EDTA (calcium independent assay) or 1 mM CaCl_2_ (calcium-dependent assay) for 30 min at 37°C. The reaction was stopped by adding the same volume of 0.1 mg/ml soybean trypsin inhibitor (T6522, Sigma, St. Louis, MO, USA) and 10 μg/ml deoxyribonuclease I (DN25, Sigma) in ice-cold DMEM, completely dissociated, and harvested at 1200× *g* for 5 min at 4 degrees. Aggregation assays were carried out in 24-well non-tissue culture plastic plates that had been precoated with 0.5% BSA/HBSS for at least 2–3 h. In each well, 10^6^ dissociated cells were mixed in 1 ml of HBSS containing 0.5% (w/v) BSA, 1 μg/ml deoxyribonuclease I, and rotated at room temperature. The reaction was stopped by adding 1 ml of 4% (w/v) paraformaldehyde (PBS), and wells were imaged. The ratio of total aggregates and total cells was measured in 10 areas per well.

### Immunochemistry and Analysis

For immunostaining, neuronal cultures were fixed with 4% (w/v) PBS for 30 min at 37°C, permeabilized with 0.1% (w/v) TritonX-100/PBS for 5 min at room temperature and blocked with 5% (w/v) skim milk/PBS for 30 min at room temperature. Cells were then incubated successively with primary and secondary antibodies. After rinsing with PBS, coverslips were mounted in Fluoro-Gel (Electron Microscopy Sciences, Hatfield, PA, USA) and imaged with a Zeiss Meta510 confocal microscope. Images were processed with Adobe Photoshop, and Image-J (Version 1.47d, Fiji).

Presynaptic differentiation (“Presynaptic” in figures) was quantified by counting separated synapsin I-positive puncta on each cell. Neurite length was measured after tracing individual neurites using Simple Neurite Tracer-3.1.3 (Longair et al., 2011; source code is available from GitHub: https://github.com/fiji/Simple_Neurite_Tracer/). Neurite ramification (“Branch”) was counted when the neurite branched at least once in apposition to a 293 cell.

For immunostaining of human Cdh2 protein, 293 cells plated on glass coverslips were fixed with 4% (w/v) PBS for 30 min at 4°C, permeabilized with 0.1% (w/v) Triton X-100 for 5 min at room temperature, microwaved in IHC-Tek epitope retrieval solution (IHC World, Ellicott City, MD, USA) for 1 min, rinsed with PBS, blocked with 5% (w/v) skim milk/PBS for 30 min, and incubated with 1:100 dilution of a rabbit monoclonal antibody EPR1791-4 (Abcam, Cambridge, MA, USA) in the Renoir Red diluent (Biocare Medical, Pacheco, CA, USA) at 4°C overnight. The cells were then incubated with anti-rabbit secondary antibodies and Neurotrace 640 (Invitrogen).

To measure transfected Cdh2 protein, the bound antibodies (NCD2) were quantified using a colorimetric enzyme-linked immunosorbent assay (ELISA). Briefly, after incubating live cells with appropriate antibodies, the PBS-fixed cells were treated with 0.3% H_2_O_2_/PBS for 30 min at room temperature, blocked with 5% (w/v) skim milk (BioRad)/PBS for 30 min, incubated with peroxidase-conjugated goat anti-rat IgG (Jackson ImmunoResearch, 1:1000 dilution in 0.5% BSA/PBS) for 2 h, rinsed with PBS, and developed with *o-*phenylenediamine/H_2_O_2_.

Other primary antibodies used in this study were as follows. Rabbit antibodies to synapsin I (Millipore, catalog #AB1543P), mouse antibody to NLGN1 (NeuroMab, Davis, CA, USA, clone# N97A/31), mouse anti-NRXN (NeuroMab, clone# N170A-1), mouse anti-MAGUK/PDZ (so-called “pan-PDZ”; NeuroMab, clone#28-86), rabbit anti-MAP2 (Santa Cruz, catalog#sc-20172), mouse anti-PSD95 (ThermoFisher/Invitrogen, clone# 7E3-1B8), rat anti-chicken Cdh2 (ThermoFisher/Invitrogen or in-house, clone# NCD2), rat anti-HA tag (Roche, clone# 3F10), mouse anti-γ-catenin (BD Transduction Labs, San Jose, CA, USA, clone#15). All the fluorophore-conjugated secondary antibodies were obtained from Jackson ImmunoResearch (West Grove, PA, USA). Acti-stain 488 phalloidin (Cytoskeleton, Inc., Denver, CO, USA) and Neurotrace 640 (ThermoFisher/Invitrogen) were used for counterstaining.

### Statistical Methods

*T*-tests, ANOVA and Tukey tests were used as appropriate. For *t*-tests, we used the *T*-TEST function in Microsoft Excel for Mac 2011 (version 14.3.1). One-way ANOVA (aov) and Tukey multiple pairwise-comparisons (TukeyHSD) were performed using R 3.4.4 for MacOS X GUI 1.70 (The R foundation)^2^.

## Results

### Generation of a Cdh2-Deficient Cell Line

**Figure 1:**
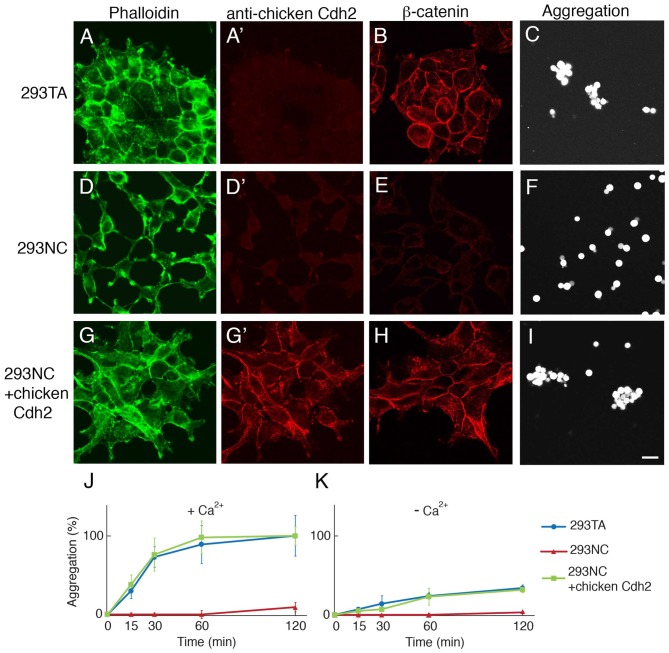
CRISPR-mediated knock-out of *cdh2* from 293TA cells.** (A–C)** Parental 293TA cells proliferate as clusters since cadherin-mediated lateral adhesion is active as revealed by phalloidin-stained cytoskeletal actin **(A)**. These human cells were not stained with anti-chicken Cdh2 antibody **(A′)** but did express human Cdh2 (see Supplementary Figure S1E). β-catenin, a cadherin-associate cytosolic protein, is concentrated at cell contacts **(B)**. After dissociation and incubation for 30 min, 293TA cells form aggregates **(C)**. **(D–F)** 293NC cells, generated by mutation of both alleles of the *cdh2* gene. 293NC cells grew as isolated cells with minimal spreading **(D)**. They lost immunoreactivity for β-catenin **(E)** and failed to aggregate **(F)**. **(G–I)** 293NC cells transfected with a full-length chicken Cdh2 construct recovered lateral adhesions **(G)**. Chicken N-cadherin accumulated at cell-cell contacts **(D′,G′)**, β-catenin was concentrated at the cell surface **(H)** and aggregation was rescued **(I)**. Bar, 20 μm. **(J, K)** Quantification of cell aggregation in the presence **(J)** or absence **(K)** of extracellular calcium (1 mM), from experiments such as those shown in (**C,F,I**; Mean ± SEM, *n* = 10). Significance of differences at 60 min and 120 min, *p* < 0.005 by Student’s *t*-test between 293TA and 293NC, and 293NC and 293NC+chicken Cdh2 in **(J)**, and *p* < 0.01 between 293TA and 293NC, and 293NC and 293NC+chicken Cdh2 in **(K)**.

We obtained HEK 293T cells from the ATCC and isolated a clone, 293TA, which grew rapidly and could be transfected efficiently. 293TA cells resembled parental HEK293T cells in that they adhered tightly to culture dishes and formed lateral adhesions at which Cdh2, actin and β-catenin were concentrated (Figures 1A,B). Following dissociation, they exhibited strong calcium-dependent aggregation (Figures 1C,J,K).

To inactivate the *cdh2* gene, we transfected 293TA cells with a plasmid encoding Cas9 and a sgRNA targeted to the amino terminus of cdh2, isolated clones, and sequenced regions surrounding the sgRNA target. One clone, called 293NC bore deletions of 10bp and 94bp on the two *cdh2* alleles, both of which led to deletion of the translation initiation site and generation of putative null alleles (Supplementary Figure S1). 293NC cells were Cdh2-negative by immunohistochemistry and exhibited markedly reduced levels of membrane associated actin and β-catenin (Figures 1D,E). Moreover, 293NC cells aggregated poorly in the presence or absence of calcium (Figures 1F,J,K). Introduction of chicken Cdh2 using transposon-aided integration (see “Materials and Methods” section) restored the localization of β-catenin and actin as well as the aggregation properties of 293NC cells to those of the parental 293TA cells (Figures 1G–K), demonstrating that their loss was due to mutation of *cdh2* rather than clonal variation or off-target effects of CRISPR editing.

### Cdh2 Promotes Neurite Branching

**Figure 2:**
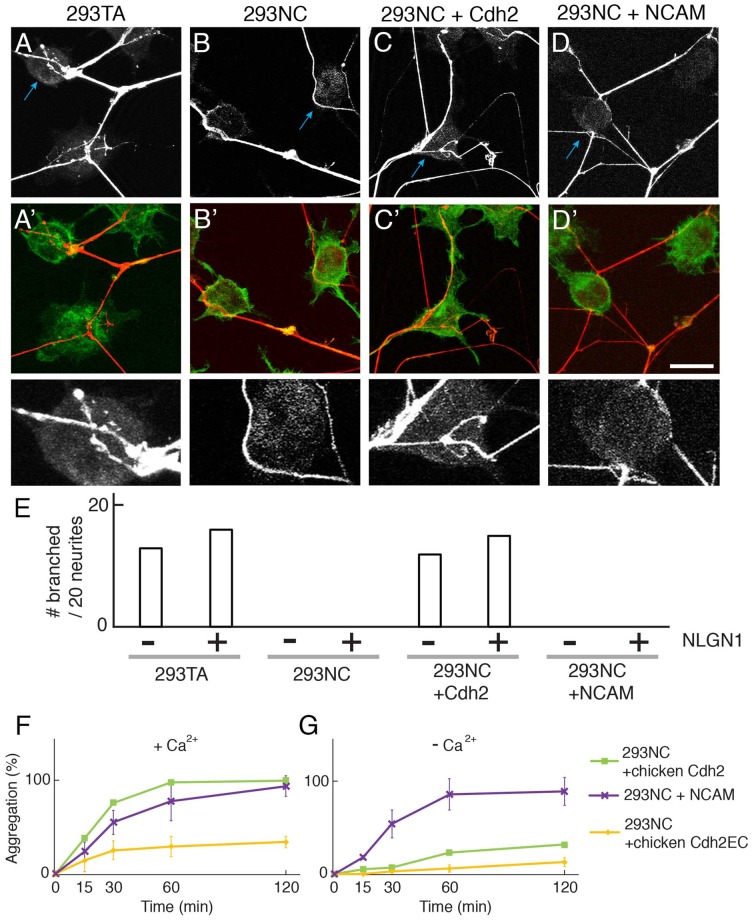
Cdh2 on 293TA cells provides favorable surface for neurite branching of cocultured cortical neurons. **(A–D,A′–D′)** Mouse cortical neurons were cultured with 293T derivatives and stained with anti-synapsin I (red) and phalloidin (green). **(A)** Cortical neurites frequently branch on 293TA cells as shown in the high-power picture (bottom; region indicated by blue arrow in top panel). **(B)** Neurites generally grew unbranched near the edge of 293NC cells **(C)** Neurites branched on Cdh2-overexpressing 293NC **(D)** Neurites failed to branch on neural cell adhesion molecule (NCAM)-overexpressing 293NC cells. Bar, 25 μm for **(A–D)**, and 5 μm for high-power images (bottom). **(E)** Percent of neurons exhibiting branching at sites of apposition with 293 cells (*n* = 20 neurons per condition). Co-expression of NLGN1 had no effect on branching in the presence or absence of Cdh2. **(F,G)** Quantification of calcium-dependent **(F)** and independent **(G)** cell aggregation assays (Mean ± SEM, *n* = 10). Significance of differences at 60 min and 120 min, *p* < 0.005 by Student’s *t*-test between 293NC and 293NC+NCAM in **(F,G)**; *p* < 0.05 between 293NC and 293NC+Cdh2EC in **(F)**; *p* < 0.05 between 293NC+Cdh2 and 293 + Cdh2EC in **(F,G)**; *p* > 0.1 between 293NC and 293NC+Cdh2EC in **(G)**. Data on 293NC and 293NC+Cdh2 cells replotted from Figure 1. 293NC cells transfected with mouse NCAM showed calcium-independent cell aggregation. 293NC cells transfected with chicken Cdh2EC showed calcium-dependent cell aggregation.

To assess effects of Cdh2 on neurite growth, we cultured embryonic day (E) 17 mouse cortical neurons with 293TA or 293NC cells. Neurites contacting 293TA cells generally branched on their surface, whereas neurites contacting 293NC cells seldom branched (Figures 2A,B,E). Introduction of Cdh2–293NC cells restored their ability to promote branching. Similar results were obtained with hippocampal neurons from postnatal day 1 rat pups (see below). Thus, Cdh2, which is known to promote neurite extension from cultured neurons (see “Discussion” section), also supports neurite branching.

To ask whether this effect is specific to Cdh2, we transfected 293NC cells with a construct encoding the calcium-independent NCAM which promotes neurite outgrowth when coated on culture dishes and is present on neuronal surfaces (e.g., Kleene et al., 2010). NCAM promoted aggregation of 293NC cells to a similar extent as Cdh2 in calcium-containing medium, although NCAM-dependent aggregation was calcium-independent (Figures 2F,G). However, NCAM did not support neurite branching (Figures 2D,E). Together these results suggest that adhesion alone is insufficient to support neurite branching.

### Cdh2 Enables Neuroligin-Dependent Presynaptic Differentiation

**Figure 3:**
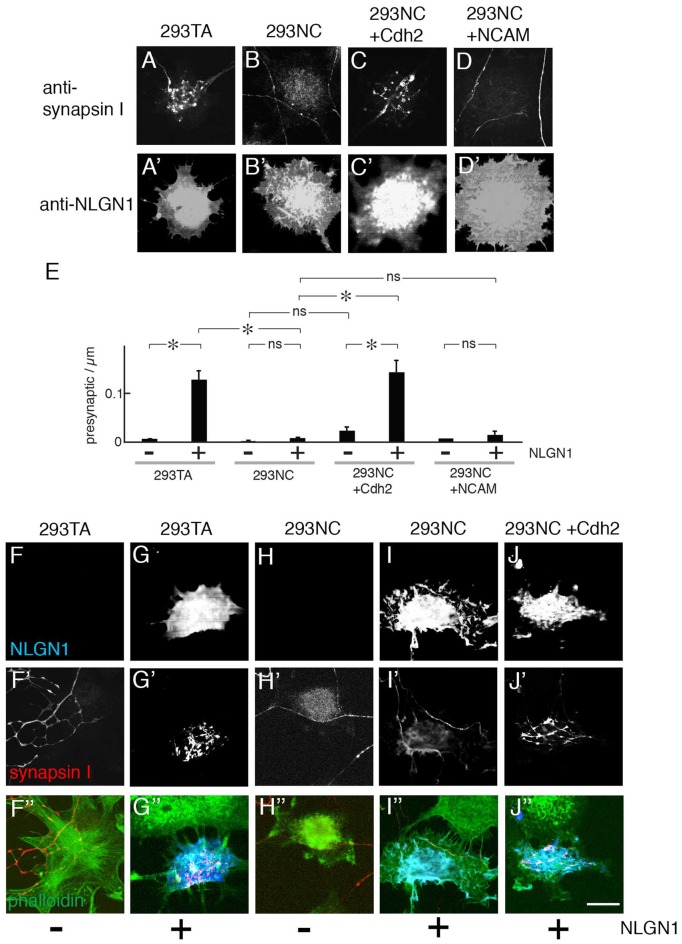
Presynaptic differentiation of neurons on NLGN1-transfected heterologous cells. **(A–D)** Mouse cortical neurons were cultured with 293TA cells and derivatives that had been transfected with NLGN1. Cultures were and stained with anti-synapsin I **(A–D)** and anti-NLGN1 **(A′–D′)**. Neurites branched and accumulated synapsin I-positive puncta when the 293 cells expressed Cdh2 **(A,C)**. **(E)** Number of synapsin-rich puncta per micron neurite-293 cell apposition, measured from images such as those in (**A–D**; Mean ± SEM, *n* = 12–17). *F*_(7,120)_ = 4.9, Pr(>F) = 7.0e-05 by one-way ANOVA followed by Tukey multiple pairwise-comparisons (*p < 0.03; ns, *p* > 0.5). **(F–J,F′–J′,F″–J″)** Hippocampal neurons from P1 rat pups were cultured on 293TA cells and derivatives as in **(A–D)**. Cultures were stained with antibodies to synapsin (red), neuroligin (NLGN; blue) and phalloidin (green). Bar, 20 μm. In **(A,C,G′,J′)**, gain was adjusted to show high-density puncta of synapsin on 293 cells; at higher gain, lower levels of synapsin immunoreactivity were visible throughout the axon, as shown in Figure 2.

Previous studies demonstrated that introduction of NLGN1 to heterologous cells enables them to promote presynaptic differentiation in co-cultured neurons, as ascertained by accumulation of puncta rich in presynaptic markers such as synapsin I (Scheiffele et al., 2000; Biederer and Scheiffele, 2007; Tsetsenis et al., 2014). We confirmed this activity using 293TA cells and Cdh2-rescued 293NC cells. In contrast, NLGN1 was unable to promote presynaptic differentiation in 293NC cells, or 293NC cells expressing NCAM (Figures 3A–D). NLGN1 had no effect on neurite branching either in the absence or presence of Cdh2 (Figure 2E). The difference in presynaptic differentiation between Cdh2-expressing and nonexpressing 293 cells was not a trivial consequence of the failure of neurite branching, in that the defect was highly significant even when the number of presynaptic puncta was normalized to the length of neurite in juxtaposition to the heterologous cell (Figure 3E). Because many previous studies on the ability of NLGN to promote presynaptic differentiation used hippocampal neurons cultured from neonatal rats, we repeated the experiments using these cells. Results for hippocampal neurons were similar to those for cortical neurons: NLGN1 expressed in 293 cells promoted presynaptic differentiation only when the 293 cells also expressed Cdh2 (Figures 3F–J).

### Cdh2-Dependent Neurite Branching and Presynaptic Differentiation Require Intracellular Interactions

**Figure 4:**
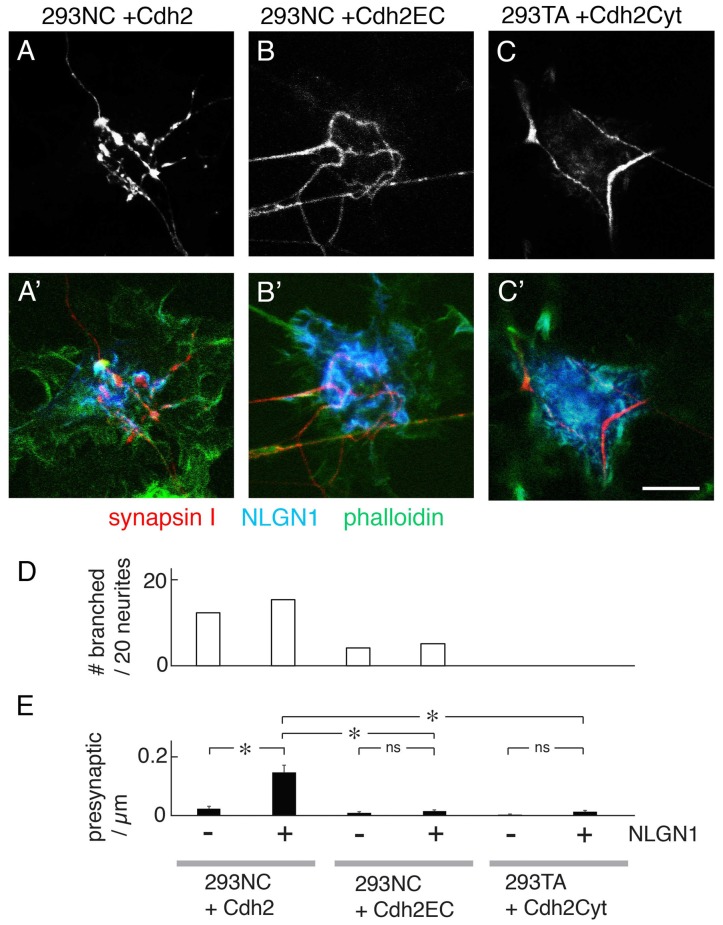
Effects of truncated Cdh2 proteins on neurite branching, but not NLGN1-mediated presynaptic differentiation. **(A–C,A′–C′)** Mouse cortical neurons were cultured on 293NC plus a full-length chicken Cdh2 **(A)**, 293NC cells expressing Cdh2EC lacking the Cdh2 cytoplasmic domain **(B)**, or 293TA cells expressing Cdh2Cyto lacking its extracellular domain **(C)**. In all cases, the cells co-expressed NLGN1. Cultures were stained with anti-synapsin I (red), anti-NLGN1 (blue), and phalloidin (green). Cdh2EC supports branching and differentiation weakly. Cdh2Cyt inhibits branching and differentiation promoted by Cdh2. Bar, 10 μm. **(D)** Percent of neurons exhibiting branching at sites of apposition with 293 cells measured from images such as those in (**A–C**; *n* = 20 neurons per condition). **(E)** Number of synapsin-rich puncta per micron neurite-293 cell apposition, measured from images such as those in (**A–C**; Mean ± SEM, *n* = 8–27). *F*_(5,87)_ = 19, Pr(>F) = 1.2e-12 by one-way ANOVA followed by Tukey multiple pairwise-comparisons (*p < 0.0001; ns, *p* > 0.5). Bar, 10 μm.

To ask whether the ability of Cdh2 to promote neurite branching or presynaptic differentiation requires intracellular Cdh2 interactions we transfected 293NC cells with a construct encoding a Cdh2 mutant (Cdh2EC) that retaining the extracellular domain but lacked the cytoplasmic domain (Levine et al., 1994). This construct was effectively expressed (Supplementary Figure S2) and restored the ability of 293NC cells to aggregate in a calcium-dependent manner, albeit less strongly than full-length Cdh2 (Figure 2F). It did not, however, restore the ability of 293NC cells to support neurite branching or NLGN1-dependent presynaptic differentiation (Figures 4A,B,D,E). We also tested a Cdh2 mutant comprising the cytoplasmic and transmembrane domains but no ectodomain (Cdh2Cyt). This mutant exerts a dominant-negative effect (Kintner, 1992) and also decreases the membrane-associated pool of Cdh2 through effects on post-transcriptional processing and transport (Nieman et al., 1999; Ozawa and Kobayashi, 2014). Expression of Cdh2Cyt abolished the ability of 293TA cells to promote neurite branching or enable NLGN1-dependent presynaptic differentiation (Figures 4C–E).

**Figure 5:**
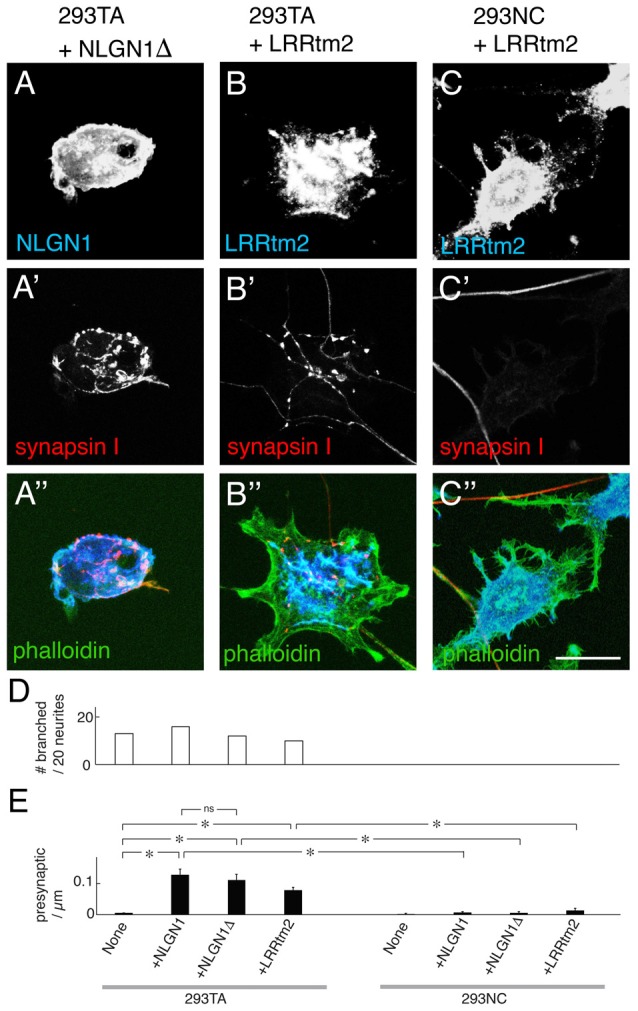
Presynaptic differentiation elicited by truncated NLGN and leucine-rich repeat transmembrane protein 2 (LRRtm2). **(A–C)** Mouse cortical neurons were cultured on 293TA cells transfected with a truncated NLGN1 lacking its PDZ-binding motif (NLGN1Δ; **A**) or LRRtm2 **(B)** or 293NC cells transfected with LRRtm2 **(C)**. Cultures were stained for NLGN1 **(A)** or LRRtm2 **(B,C)** plus synapsin I (red, **A′–C′**) and actin (green, **A″–C″**). Bar, 20 μm. **(D)** Percent of neurons exhibiting branching at sites of apposition with 293 cells measured from images such as those in (**A–C**; *n* = 20 neurons per condition). **(E)** Number of synapsin-rich puncta per micron neurite-293 cell apposition, measured from images such as those in (**A–C**; Mean ± SEM, *n* = 10–24). *F*_(7,124)_ = 17, Pr(>F) = 7.5e-16 by one-way ANOVA followed by Tukey multiple pairwise-comparisons (*p < 0.0001; ns, *p* = 0.97).

One explanation for the failure of the Cdh2EC mutant to enable presynaptic differentiation is that Cdh2 might recruit NLGN1 to a scaffold by means of interactions with scaffolding proteins comprising PDZ domains (Irie et al., 1997; discussed in Yamagata and Sanes, 2010). To test this possibility, we co-transfected 293 cells with a NLGN1 construct lacking the carboxy-terminal tripeptide known to mediate interaction with PDZ proteins. The mutant NLGN1 promoted presynaptic differentiation as well as wild-type NLGN1 (Figures 5A,D). Thus, the Cdh2 cytoplasmic domain does not enable NLGN1-dependent presynaptic interaction by a PDZ-dependent scaffolding mechanism. An attractive alternative is that interaction of Cdh2 with the 293 cell cytoskeleton enables it to form cluster at sites of contact with neurites, which in turn strengthens or stabilizes the cell-cell interaction.

### Cdh2 Enables LRRtm2- and Cadm1-Dependent Presynaptic Differentiation

To ask whether the ability of Cdh2 to enable presynaptic differentiation is restricted to NLGNs, we tested the leucine-rich repeat transmembrane protein 2 (LRRtm2) and the immunoglobulin superfamily cell adhesion molecule (Cadm1, also called Syncam1, Igsf4, Necl-2 and Tslc-1), which, have also been shown to promote presynaptic organizer when expressed in heterologous cells (Biederer et al., 2002; de Wit et al., 2009; Ko et al., 2009; Linhoff et al., 2009; Siddiqui et al., 2010). LRRtm2 was as effective as NLGL1 in promoting presynaptic differentiation in 293TA cells but was incapable of doing so in 293NC cells (Figures 5B–D). Similar results were obtained with Cadm1 (data not shown).

### Cdh4 and Cdh6 Promote Neurite Branching and Enable Presynaptic Differentiation

**Figure 6:**
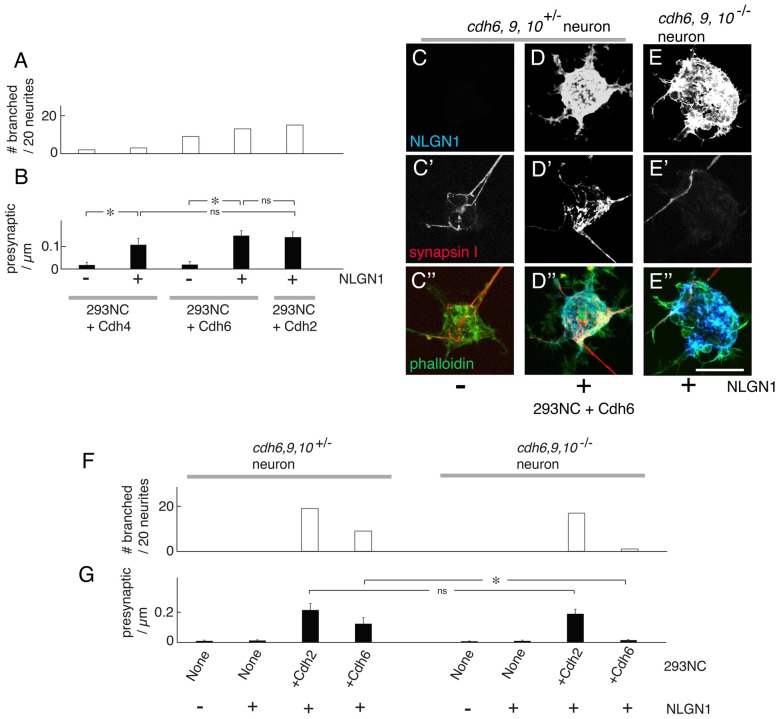
Cdh4 and Cdh6 enable NLGN1-dependent presynaptic differentiation. **(A)** Percent of neurons exhibiting branching at sites of apposition with 293NC cells expressing Cdh2, Cdh4 or Cdh6 with or without NLGN1, measured as in Figures 2, 4, 5. Data for Cdh2 replotted from Figure 2E (*n* = 20 neurons per condition). **(B)** Number of synapsin-rich puncta per micron neurite apposition to 293NC cells as in **(A)**, measured as in Figures 3–5. Data for Cdh2 replotted from Figure 3E measured from images such as those in (**A–C**; Mean ± SEM, *n* = 8). *F*_(4,35)_ = 8.6, Pr(>F) = 6.0e-05 by one-way ANOVA followed by Tukey multiple pairwise-comparisons (*p < 0.004; ns, *p* > 0.5). **(C–E)** Mouse cortical neurons were cultured on 293NC cells expressing Cdh6, with or without NLGN1. Neurons were from *cdh6/9/10*^+/−^ **(C,D)** or *cdh6/9/10*^−/−^ mice. The cultures were stained for NLGN1 **(C–E)**, synapsin **(C′–E′)** and phalloidin **(C″–E″)**. Bar, 20 μm. **(F)** Percent of neurons exhibiting branching at sites of apposition with 293 cells measured from images such as those in (**C–E**; *n* = 20 neurons per condition). **(G)** Number of synapsin-rich puncta per micron neurite-293 cell apposition, measured from images such as those in (**C–E**; Mean ± SEM, *n* = 8). Difference between *cdh6-9-10*^+/−^ and *cdh6-9-10*^−/−^ neurons on 293NC cells+Cdh6 is significant (*F*_(7,56)_ = 13, Pr(>F) = 1.5e-09 by one-way ANOVA followed by Tukey multiple pairwise-comparisons; *p = 0.0056) but difference on 293NC cells +Cdh2 cells is not (ns, *p* = 0.99).

The classical cadherins comprise Type I cadherins (including Cdh1–4) and Type II cadherins (including Cdh6–11; Hirano and Takeichi, 2012). We expressed another Type I cadherin, Cdh4, and a Type II cadherins, Cdh6, in 293NC cells and co-cultured them with cortical neurons. We chose these cadherins because they are expressed by large subsets of cortical neurons (e.g., Obst-Pernberg et al., 2001; Lefkovics et al., 2012). Cdh4 and Cdh6 were both able to promote neurite branching and enable NLGN1-dependent presynaptic differentiation. In our assay, Cdh6 was as effective as Cdh2, whereas Cdh4 was less effective (Figures 6A,B). One potential explanation is that a larger fraction of cortical neurons express *cdh6* than *cdh4*.

The ability of cadherins in 293 cells to enable neurite branching and presynaptic differentiation is likely to result from hemophilic interactions with corresponding cadherins on neurites. The efficacy of Cdh6 allowed us to test this assumption, because we recently generated mutant mice lacking Cdh6, as well as Cdh9 and Cdh10, which are capable of binding to Cdh6 (Shimoyama et al., 2000; Basu et al., 2017; Duan et al., submitted). We compared the ability of neurons from *cdh6;cdh9;cdh10* triple mutants (*cdh6/9/10*^−/−^) and littermate controls (*cdh6/9/10*^+/−^) to branch on 293NC cells transfected with Cdh2 or Cdh6, and to exhibit presynaptic differentiation on 293NC cells expressing NLGN1 plus either Cdh2 or Cdh6. Whereas wild-type neurons responded similarly to 293NC cells bearing Cdh2 or Cdh6, neurons from *cdh6/9/10* triple mutants responded to Cdh2 but not Cdh6 (Figures 6C–G,C′–E′,C″–E″). These results suggest that cadherins on target cells act by binding to matched cadherins on neurites.

### Cdh2 Enables Neurexin-Dependent Postsynaptic Differentiation

**Figure 7:**
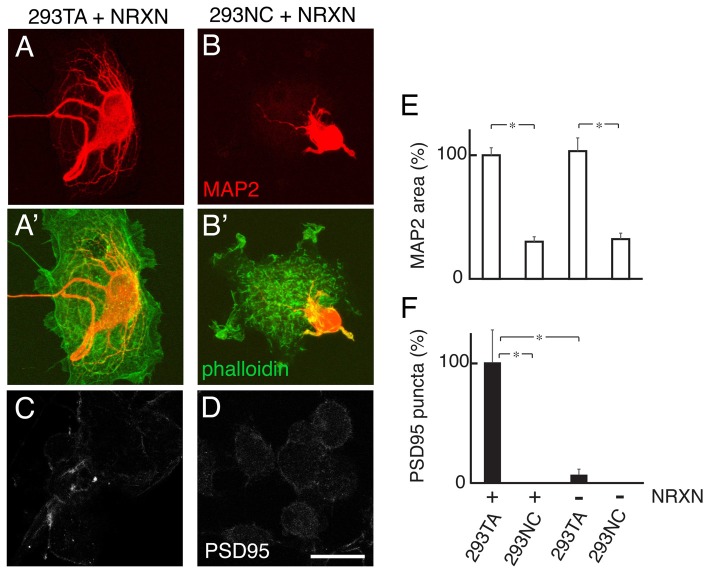
Cdh2 supports dendritic branches neurexin (NRXN)-dependent postsynaptic differentiation. **(A,B)** Rat hippocampal neurons were cultured with 293TA **(A)** or 293NC **(B)** cells transfected with NRXN, then stained with anti-MAP2 to visualize dendrites. 293 cells were visualized by phalloidin staining (green in **A′,B′**). **(C,D)** Immunostaining for PSD95 on NRXN-transfected 293TA **(C)** or 293NC cells **(D)**. Bar, 20 μm. **(E,F)** Quantification of MAP2-positive protrusions (relative areas) **(E)** and PSD95 punta **(F)**. *F*_(3,36)_ = 13, Pr(>F) = 8.2e-06 by one-way ANOVA followed by Tukey multiple pairwise-comparisons; *p < 0.0002.

Previous studies showed that heterologous (COS) cells expressing NRXNs, presynaptically-concentrated binding partners of NLGNs, can promote postsynaptic differentiation when cultured with rat hippocampal neurons, as judged by accumulation of puncta rich in PSD95, a component of excitatory postsynaptic densities (Graf et al., 2004). To ask whether this effect requires Cdh2, we cultured hippocampal neurons with 293TA or 293NC cells transfected with NRXN1β. We marked dendrites by staining with antibodies to the dendritically concentrated microtubule-associated protein MAP2 and used anti-PSD95 to mark postsynaptic densities. Dendritic branching was more robust at sites of contact with 293TA cells than at sites of contact with 293NC cells. Thus, dendritic branching, like axonal branching, is promoted by Cdh2. Moreover, the ability of NRXN1β to promote postsynaptic differentiation was several-fold greater in the presence of Cdh2 (293TA cells) than in its absence (293NC cells; Figure 7).

## Discussion

The HEK293 cell line, derived from a HEK cell, is frequently used to analyze cellular mechanisms, either by exploiting its complement of endogenous constituents or by introducing cDNAs to express proteins of interest; they are also used to produce recombinant proteins for therapeutic and investigative purposes. Their popularity (the search terms “HEK293” or “HEK 293” retrieves over 48,000 citations on Pubmed) is in large part because they are easy to grow and transfect. As tools for analysis of synaptic interactions, however, the fact that they express *cdh2* is disadvantageous in two respects. First, Cdh2 renders them prone to aggregation, limiting their use for assessing adhesion mediated by synaptic adhesion and recognition molecules. Second, as demonstrated here, endogenous *cdh2* expression prevented us from using them to analyze roles of cadherins in synaptogenesis.

To circumvent these limitations, we generated a subclone, 293TA, and then used CRISPR/Cas9-mediated mutagenesis to inactivate both *cdh2* alleles in this subclone, generating the Cdh2-deficient line we call 293NC. The 293NC line retained the growth properties and transfection efficiency of the parental line. Loss of cell surface-associated catenin and of calcium-dependent aggregation in 293NC cells, along with the ability to rescue these properties by re-introducing Cdh2, suggests that Cdh2 is the principal classical cadherin expressed by the parental cells, although it is possible that they also express low levels of other family members.

In a recent study, we exploited the decreased adhesivity of 293NC cells to assay adhesion mediated by wild-type and mutant immunoglobulin superfamily molecules (Goodman et al., 2016; description of the line was deferred to the present manuscript). Here, we used 293NC cells to analyze roles of cadherins in guiding the growth and synaptic differentiation of neurites.

We report five main results. First, when Cdh2 is displayed on the surface of heterologous cells, it promotes branching of neurites. Several previous studies documented the ability of N-cadherin to promote neurite outgrowth (Matsunaga et al., 1988; Tomaselli et al., 1988; Bixby and Zhang, 1990; Payne et al., 1992), either in cell-associated form or coated on substrates but did not discuss effects on branching. However, we found that Cdh2 promoted terminal branching of retinal ganglion cell axons in retinorecipient laminae of chick optic tectum; blocking antibodies decreased branching and led to axons growing beyond their normal termination zone (Inoue and Sanes, 1997). 293NC cells may provide a useful system for analyzing the mechanism by which cadherins affect branching.

Second, Cdh2 enabled differentiation by four different synaptic organizers, NLGN1, NRXN1β, LRRtm2 and Cadm1. NLGN1, LRRtm2 and Cadm1, which are concentrated in postsynaptic membranes *in vivo*, promoted clustering of vesicles in apposed axons when Cdh2 was present but not when it was absent. Likewise, NRXN1β, which is concentrated presynaptic membranes *in vivo*, promoted aggregation of PSD95 in apposed dendrites in the presence but not the absence of Cdh2. Two previous studies used Cdh2-deficient neurons to show cooperation between Cdh2 and NLGN1 in presynaptic differentiation (Stan et al., 2010; Aiga et al., 2011). Interactions between Cdh2 and NRXN1β or LRRtm2 have not, to our knowledge, been reported previously. Thus, our study demonstrates that Cdh2 cooperates with multiple synaptic organizers.

This conclusion appears to be at odds with previous studies showing that beads coated with NLGN1, LRRtm2 or NRXN1 are able to elicit synaptic differentiation when applied to cultured neurons (Dean et al., 2003; Graf et al., 2004; Linhoff et al., 2009). However, our model is that cadherin strengthens the adhesion of neurites to their synaptic partners (see below). In the case of beads, the high density of the synaptic organizer and/or a high charge on the bead (Burry, 1980; Peng et al., 1981) may provide the adhesive strength that is generated *in vivo* by homophilic cadherin interactions at cell-cell contacts.

Third, in contrast to its ability to enable organizer-dependent synaptic differentiation, Cdh2 promoted minimal synaptic differentiation on its own. Previous studies involving mutation, down-regulation or blockade of Cdh2 in neurons (Bozdagi et al., 2000, 2004; Togashi et al., 2002; Jüngling et al., 2006; Kadowaki et al., 2007; Stan et al., 2010; Aiga et al., 2011) were unable to address this issue, because they could not distinguish between a direct effect of Cdh2 or cooperation with other organizing molecules likely present in the cells. More recently, however, Flannery and Brusés (2012) used heterologous cell cultures of brainstem cholinergic neurons and CHO cells, which lack Cdh2, and documented Cdh2-dependent differentiation of cholinergic presynaptic terminals. The apparent difference between our results and theirs might reflect cell type-specific differences in responses to Cdh2 or perhaps the presence of endogenously-expressed synaptic organizers in CHO cells.

Fourth, Type II classical cadherins as well as Cdh2 (a Type I classical cadherin) are able to promote neurite branching and enable NLGN1-dependent presynaptic differentiation. Previous studies have demonstrated roles of Type II cadherins in specifying connectivity between appropriately matched partners, and documented loss of functional synapses in their absence (Suzuki et al., 2007; Duan et al., 2014; Kuwako et al., 2014; Basu et al., 2017). We suggest that these phenotypes reflect two distinct mechanisms of cadherin action: selective adhesion, leading to appropriate connectivity, and cooperation with synaptic organizers, enabling synaptic function.

Fifth, our results suggest that synaptic roles of cadherins require matching cadherins in both synaptic partners. This conclusion is based on experiments in which we used neurons from a mouse mutant lacking Cdh6 as well as its alternate binding partners, Cdh9 and Cdh10 (Duan et al., submitted). Neurons from both wild-type and mutant mice exhibited robust presynaptic differentiation at sites of contact with 293NC cells expressing *cdh2*, but only the wild-type cells exhibited presynaptic differentiation at sites of contact with 293NC cells expressing *cdh6*.

**Figure 8:**
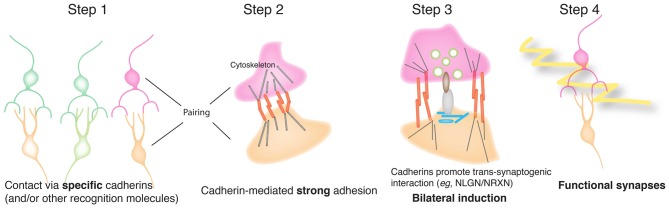
Model for cooperation of cadherins and synaptic organizers in synapse formation. In this model, classical cadherins act homophilically to mediate selective interactions between synaptic partners. Strong cadherin-dependent adhesion then promotes terminal branching and also enables productive interactions between pre- and postsynaptic organizing molecules such as NLGNs and NRXNs. The organizers then recruit cytoplasmic and cytoskeletal components to generate a functional, differentiated synapse.

Together, these results suggest a model for cooperation between cadherins and synaptic organizers in which classical cadherins are initial determinants of synaptic specificity, and facilitators of trans-synaptic signaling by synaptic organizers (Figure 8). The cadherin might enable synaptic organizers to act by complex signaling mechanism, as shown here by the inability of a Cdh2 truncated construct (Cdh2EC) or NCAM to enable NLGN1-mediated presynaptic differentiation. A simpler alternative, however, is that cadherins mediate strong enough adhesion between synaptic partners for pre- and postsynaptic organizers to engage productively and do so based on their well-studied ability to mobilize the cytoskeleton (Gumbiner, 2005; Hirano and Takeichi, 2012; Leckband and de Rooij, 2014).

## Author Contributions

MY performed all the experiments. XD generated and characterized cdh6/9/10 triple mutant mice. MY and JRS designed experiments, analyzed data and wrote the article. All authors reviewed the manuscript.

## Conflict of Interest Statement

The authors declare that the research was conducted in the absence of any commercial or financial relationships that could be construed as a potential conflict of interest.

## Footnotes

^1^http://iclac.org/databases/cross-contaminations/

^2^https://www.r-project.org
