# Supplementary material for: Cadherins Interact With Synaptic Organizers to Promote Synaptic Differentiation
Source: Front Mol Neurosci. 2018 Apr 30;11:142. doi: 10.3389/fnmol.2018.00142 (PMC5936767; doi:10.3389/fnmol.2018.00142)
Supplement: Supplementary file 1 [file Image_1.PDF]

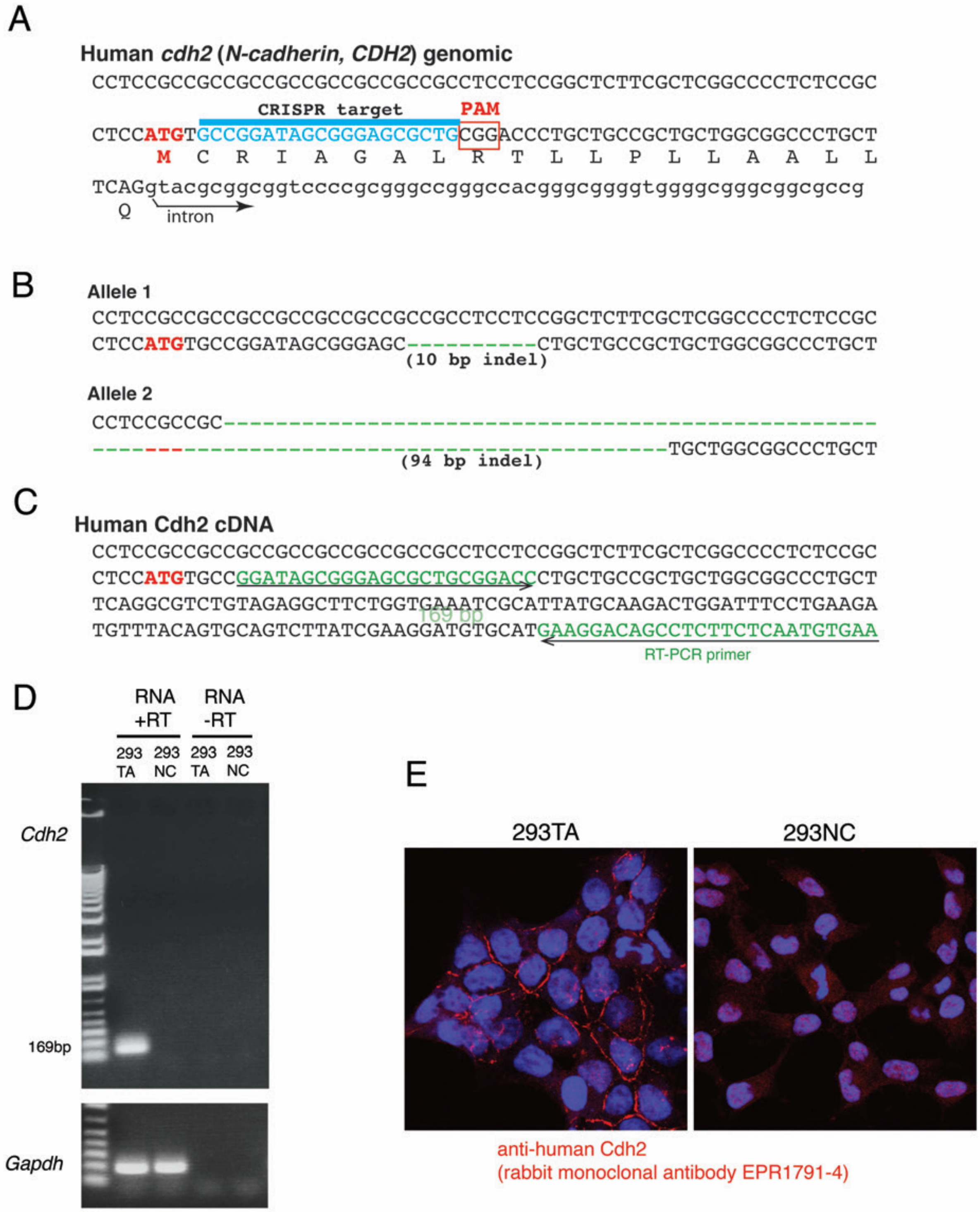

FIGURE S1. Disruption of human *cdh2* genes in 293T cells.

(A) N-terminal portion of the human *cdh2* gene showing position of the sgRNA used for Cas9-mediated mutagenesis. The protospacer adjacent motif (PAM) is boxed.

(B) Indels of 10bp and 94bp detected by sequencing the *cdh2* gene in 293NC cells. These mutations inactivate the *cdh2* gene by affecting translation due to a frame shift (allele 1) and by deleting the translational initiation site (allele 2).

(C) PCR primer sequences used to verify deletion of sequences in *cdh2* mRNA in 293NC.

(D) RT-PCR of RNA isolated from 293TA and 293NC cells using primers in "C" Expression of *Gapdh* is similar between 293TA and 293NC, and the PCR products were not detected when reverse transcriptase (RT) was omitted from the RT-PCR reaction, excluding the possibility of amplification from genomic DNA.

(E) 293TA and 293NC cells were immunostained with a monoclonal antibody to human Cdh2 (EPR1791-4). Endogenous human Cdh2 is concentrated at cell-cell contacts in 293TA, but was not observed in 293NC. Bar, 20μm.

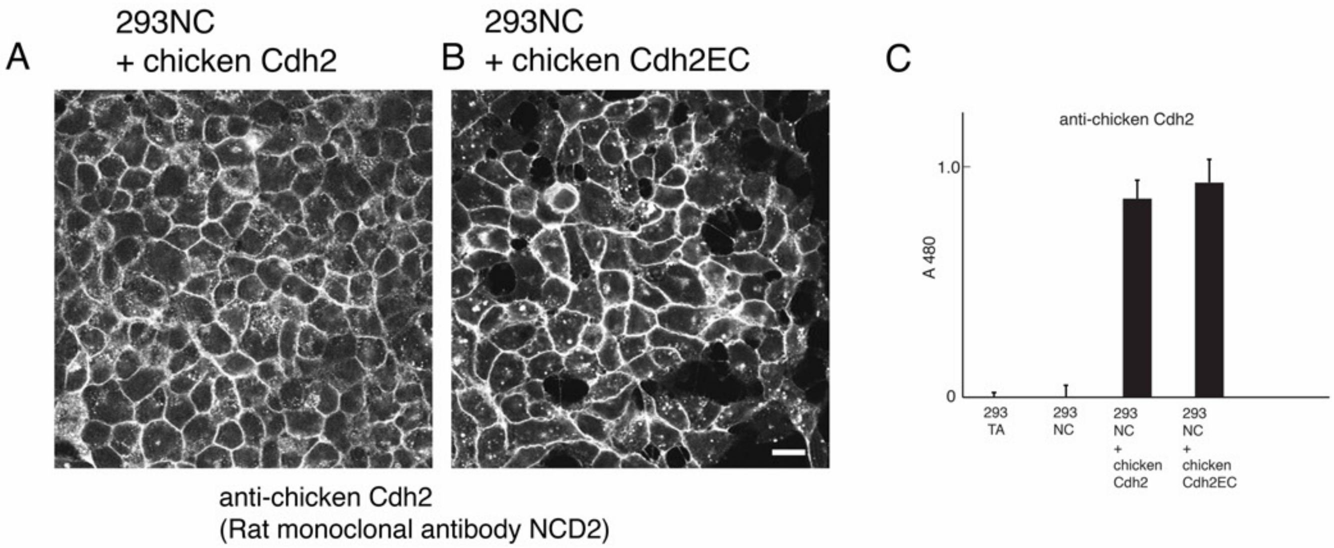

FIGURE S2. Effect of transfected Cdh2 and relevant constructs.

(A, B) 293NC cells were transfected with a full length chicken Cdh2 cDNA (A) or chicken Cdh2EC (B) and stained with anti-chicken Cdh2 antibody (NCD2). Bar, 20μm.

(C) Colorimetric enzyme-linked immunosorbent assay (ELISA) to evaluate the expression level of transfected chicken cdh2 in each cell line (Mean ± SEM, n=5).
